# Identification of mitochondria-related biomarkers in liver fibrosis via interpretable machine learning and WGCNA: transcriptomic analysis and *In Vivo* validation

**DOI:** 10.3389/fimmu.2026.1705706

**Published:** 2026-05-28

**Authors:** Yupeng Ma, Xinhong Chen, Lujin Yin, Yongbin Chi, Denghai Zhang, Xiaocheng Xue, Xue Zhang

**Affiliations:** 1 Shanghai Health Commission Key Lab of Artificial Intelligence (AI)-Based Management of Inflammation and Chronic Diseases, Sino-French Cooperative Central Lab, Shanghai Pudong Gongli Hospital, Shanghai, China; 2 School of Gongli Hospital Medical Technology, University of Shanghai for Science and Technology, Shanghai, China; 3 Department of Otolaryngology, Shanghai Pudong Gongli Hospital, Shanghai, China

**Keywords:** biomarkers, HSC–immune axis, liver fibrosis, machine learning, mitochondrial dysfunction, WGCNA

## Abstract

**Background:**

Hepatic fibrosis is a key pathological stage in the progression of many chronic liver diseases; timely intervention is critical to preventing cirrhosis and hepatocellular carcinoma. Mitochondria regulate energy metabolism, lipid homeostasis, and redox balance, and their dysfunction is increasingly recognized as a driver of fibrogenesis.

**Objective:**

To identify key mitochondria-related genes associated with liver fibrosis and explore their mechanistic roles and therapeutic potential using a multi-omics mining strategy.

**Methods:**

Fibrosis-related bulk RNA-seq datasets (GSE152329, GSE167216, GSE119953, GSE254610) and scRNA-seq datasets (GSE145086, GSE233084) were retrieved from GEO. WGCNA-derived modules were intersected with DEGs and a mitochondrial gene set to obtain candidate genes. GO and KEGG enrichment analyses were performed with clusterProfiler, and TF activity was inferred with decoupleR. An XGBoost algorithm was utilized to prioritize critical mitochondrial targets. Cell–cell communication was analyzed using CellChat. A CCl_4_-induced C57BL/6 mouse model was established for HE/Masson staining, Western blotting, and TSA-IF. Crucially, functional validation was performed in human LX-2 hepatic stellate cells via ACOT9 knockdown to assess its regulatory role in fibrogenesis.

**Results:**

Bulk RNA-seq and WGCNA identified 38 mitochondria-related DEGs in CCl_4_-induced fibrosis. Machine learning prioritization highlighted Acot9, Aldh1b1, and Pck2 as key targets. scRNA-seq revealed specific expression patterns (predominantly Aldh1b1 in hepatocytes; Pck2 in cholangiocytes/HSCs; Acot9 in endothelial subsets), and CellChat analysis demonstrated remodeling of TGF-β and COLLAGEN signaling networks. *In vivo*, ACOT9, ALDH1B1, and PCK2 were upregulated in fibrotic liver tissue, consistent with transcriptomic changes. In the human cirrhosis dataset (GSE254610), ACOT9 was also significantly upregulated, confirming its relevance to advanced human disease. *In vitro*, silencing ACOT9 in human LX-2 cells significantly downregulated α-SMA, COL1A1, and TGF-β, indicating that ACOT9 functions as an upstream regulator of classical fibrotic markers.

**Conclusions:**

Acot9 was identified as a key mitochondrial target associated with liver fibrosis. Its consistent upregulation in fibrotic liver tissue and, notably, the ACOT9-dependent modulation of fibrosis markers in hepatic stellate cells highlight its mechanistic relevance and potential as a therapeutic target for further study.

## 1 Introduction

As the central organ for metabolism and detoxification, the liver is highly vulnerable to persistent insults caused by drug abuse, chronic alcohol consumption, viral or parasitic infections, lipid deposition, and immune imbalance. These insults collectively contribute to the progressive expansion of chronic liver disease (CLD), encompassing diverse entities such as nonalcoholic/alcoholic fatty liver disease, viral hepatitis, and autoimmune hepatitis (1). Despite their heterogeneous etiologies, CLDs share common pathological hallmarks, including persistent inflammation and excessive collagen deposition, which ultimately drive the onset and progression of hepatic fibrosis (2–4). In the absence of effective intervention, fibrosis may advance to cirrhosis, liver failure, and even hepatocellular carcinoma, leading to a marked reduction in patient survival (5, 6). Global data indicate that between 2010 and 2019, liver disease-related mortality increased from 3% to 3.5%, imposing a substantial burden on public health and healthcare systems (7). Hepatic fibrosis is a dynamic process that can be prevented or even reversed through timely removal of causal factors and appropriate therapeutic strategies; however, its therapeutic window is limited. Therefore, the identification of molecular biomarkers closely associated with fibrogenesis is of great importance for assessing disease status, guiding pharmacological decisions, and developing more precise interventions. Among the multiple mechanistic pathways implicated in liver fibrosis, mitochondria have emerged as a focal point of research due to their central role in regulating hepatocyte energy metabolism, oxidative stress, and apoptosis.

Mitochondria serve as the central hub for cellular energy metabolism and signaling regulation. In addition to orchestrating physiological processes such as fatty acid oxidation, the tricarboxylic acid (TCA) cycle, and oxidative phosphorylation, mitochondria are broadly involved in pathological processes including calcium homeostasis, apoptosis, oxidative stress, and inflammatory responses (8–10). Accumulating evidence indicates that mitochondrial dysfunction is closely associated with the pathogenesis of multiple liver diseases, including fibrosis, cirrhosis, and hepatocellular carcinoma, through mechanisms involving excessive reactive oxygen species (ROS) generation, impaired ATP production, and mitochondria-mediated programmed cell death (11–13). Notably, structural abnormalities and metabolic disturbances of mitochondria have been widely observed in key cell types such as hepatocytes and hepatic stellate cells (HSCs) during the progression of hepatic fibrosis, suggesting that mitochondria may play a pivotal role in both the initiation and advancement of fibrogenesis (14). Therefore, identifying mitochondria-associated biomarkers in liver fibrosis holds promise for uncovering critical metabolic nodes in disease progression and for developing novel targeted therapeutic strategies.

With the rapid advancement of high-throughput sequencing technologies, transcriptome sequencing has been widely applied to investigate the molecular mechanisms of liver fibrosis (15). Bulk RNA sequencing enables the capture of gene expression changes at the whole-tissue level, making it suitable for identifying differentially expressed genes and enriched pathways associated with disease progression. In contrast, single-cell RNA sequencing (scRNA-seq) provides single-cell resolution to reveal cellular heterogeneity within tissues, thereby uncovering the expression patterns of mitochondria-related genes in specific cell types and characterizing intercellular interactions. The complementary strengths of these approaches offer a multidimensional framework for elucidating the mechanisms underlying hepatic fibrosis.

In the face of large-scale and high-dimensional omics data, machine learning has emerged as a powerful tool for data mining due to its superior capability in feature recognition, and it has been widely applied to biomarker discovery and predictive modeling (16). Among various algorithms, eXtreme Gradient Boosting (XGBoost) is exceptionally suited for transcriptomic analysis owing to its robust handling of non-linear data structures and intrinsic regularization against overfitting in small-sample cohorts. However, despite these algorithmic advantages, complex ensemble models like XGBoost and random forest are often criticized as “black-box models,” as they provide limited interpretability regarding the contribution of individual features to model outputs, thereby restricting their clinical translational potential. To address this issue, interpretable algorithms such as SHAP (SHapley Additive exPlanations) have recently been developed. SHAP, based on Shapley value theory from game theory, assigns explicit contribution values to each feature for each sample, thereby quantifying both the direction and magnitude of the influence of individual variables on predictive outcomes. This approach greatly enhances the interpretability and credibility of machine learning models (17–19).

Taken together, this study integrates single-cell and bulk transcriptomic data, applying a combination of bioinformatics approaches, interpretable machine learning algorithms, and experimental validation to systematically identify key mitochondria-related genes associated with liver fibrosis and to delineate aberrant intercellular communication patterns under disease conditions. By combining computational prediction with *in vivo* and *in vitro* verification, our findings aim to provide novel mechanistic insights and identify potential therapeutic targets for the intervention of liver fibrosis.

## 2 Methods

### 2.1 Data sources and preprocessing

Liver fibrosis–related datasets were retrieved from the GEO database, including bulk transcriptome datasets GSE152329, GSE167216, and GSE119953 derived from mouse liver fibrosis models, and the GSE254610, GSE298435 dataset derived from human clinical samples.

The GSE152329 dataset, containing 198 samples from 99 mouse strains (99 controls and 99 CCl_4_-induced fibrosis samples), was generated within a single experimental batch under identical RNA extraction, library preparation, and sequencing conditions. Therefore, no batch correction was required. Differential expression analysis, weighted gene co-expression network analysis (WGCNA), and machine learning model training were conducted based on this dataset.

The GSE167216 dataset, consisting of 18 control and 18 CCl_4_-induced fibrotic samples, was used as an independent external validation cohort to evaluate the model’s generalizability.

To further verify the model’s robustness across different etiologies of liver fibrosis, an additional dataset, GSE119953, was introduced. This dataset includes control (9 samples) and multiple experimental groups—DDC, DDC+HFD, and DDC+EtOH (17 fibrotic samples in total). The DDC (3,5-diethoxycarbonyl-1,4-dihydrocollidine) model induces cholestatic fibrosis comparable to the BDL (bile duct ligation) model, whereas DDC+HFD and DDC+EtOH respectively mimic metabolic (MCD-like) and alcoholic liver fibrosis.

Additionally, two independent human transcriptomic datasets, GSE254610 (4 healthy and 6 cirrhosis samples) and a larger cohort GSE298435 (14 healthy and 17 cirrhosis samples), were included to validate the clinical relevance of the candidate targets.

To ensure comparability across these datasets, all raw count matrices were uniformly transformed into Transcripts Per Million (TPM) for normalization. For each gene, Reads Per Kilobase (RPK) were calculated as:


$$RPK=\frac{\text{Raw counts}}{\text{Transcript length }(\text{kb})}$$

For each sample, a scaling factor was obtained as the sum of all RPK values, and TPM was computed as:


$$TPM=\frac{RPK}{\text{Scaling factor}}\times 10^{6}$$

This normalization approach corrects for both gene length and sequencing depth, providing consistent expression scales across datasets for integrative model validation (20, 21).

Single-cell RNA sequencing (scRNA-seq) datasets were obtained from GSE145086 and GSE233084, both generated using the 10X Genomics platform (22, 23).

Single-cell analyses were performed using Seurat v5. Comprehensive quality-control (QC) filtering was applied prior to downstream analysis. Cells with fewer than 200 detected genes (nFeature_RNA < 200) were removed to exclude low-quality or empty droplets. Cells with mitochondrial gene proportions exceeding 15% were excluded to remove stressed or dying cells, and those with erythrocyte-associated gene content greater than 1% were also discarded. Doublets were identified using a cluster-level marker co-expression strategy: clusters displaying simultaneous expression of canonical markers from two biologically distinct hepatic lineages were considered putative doublet-enriched populations. Based on this criterion, clusters 10, 12, 14, 15, 17, 18, and 19 exhibited mixed or ambiguous marker profiles and were removed prior to subsequent analyses.

Following QC, raw counts were normalized using the NormalizeData function, highly variable genes were identified with FindVariableFeatures, and the data were scaled with ScaleData. Principal component analysis (PCA) was applied for dimensionality reduction, and batch effects across samples were corrected using the Harmony algorithm. Sample-level metadata was provided to Harmony as the batch variable; the eight samples comprised four CCl_4_-treated samples (CCl4_1: 6242 cells; CCl4_2: 6505 cells; CCl4_3: 6681 cells; CCl4_4: 6252 cells) and four vehicle controls (Vehicle1: 5399 cells; Vehicle2: 3988 cells; Vehicle3: 7700 cells; Vehicle4: 5027 cells). A shared nearest-neighbor graph was then constructed, clustering was performed at a resolution of 0.5, and the results were visualized using UMAP. Final cell-type annotations were assigned on the basis of canonical marker genes and supporting evidence from published literature (**Table 1**).

**Table 1:** Marker genes used for cell-type annotation in the single-cell RNA-seq analysis.

Cell type	Marker genes
LSEC (Liver sinusoidal endothelial cells)	Kdr, Clec4g, Stab2, Fcgr2b
qHSC (Quiescent hepatic stellate cells)	Lrat, Reln, Des
Kupffer cells	Adgre1, Vsig4, Csf1r, C1qa, C1qc, Cybb, Cd68, Mpeg1
actHSC (Activated hepatic stellate cells)	Col1a1, Col1a2, Mmp2
Ly6a^+^ EC (Ly6a^+^ endothelial cells)	Ly6a, Cldn5, Tm4sf1
CV EC (Central vein endothelial cells)	Rspo3, Wnt2
Hepatocytes	Bhmt, Gnmt, Apoa1
Cholangiocytes	Epcam, Tm4sf4, Cdh1, Krt19
Mesothelial cells	Msln, Upk1b, Upk3b, Rspo1
Capsular fibroblasts	Cdh11, Saa3, Igfbp6, Nkain4
B cells	Ighm, Cd79a
Proliferating cells (cycling cells)	Mki67, Top2a, Birc5

### 2.2 Differential expression analysis

Differential expression analysis was performed using the limma package. The screening thresholds were set as adjusted *p* < 0.05 and |logFC| > 2. Genes with logFC > 2 were defined as upregulated, while those with logFC < –2 were defined as downregulated. The results of differential expression analysis were visualized using volcano plots generated with the ggplot2 package.

### 2.3 Weighted gene co-expression network analysis

In this study, weighted gene co-expression network analysis (WGCNA) was performed using the WGCNA package in R (24). First, sample hierarchical clustering was performed to detect potential outliers. Based on the sample dendrogram, a specific cutHeight threshold of 100 was applied, resulting in the exclusion of one outlier sample. Consequently, exactly 197 high-quality samples were retained to ensure the robustness of subsequent analyses. Next, an appropriate soft-thresholding power (power = 12) was selected to construct an adjacency matrix based on the topological overlap matrix (TOM). Genes with similar expression patterns were then grouped into distinct modules using average linkage hierarchical clustering according to TOM-based similarity. Furthermore, modules showing significant positive or negative correlations with clinical traits were identified. Module membership (MM) and gene significance (GS) values were calculated to evaluate the strength of association between genes, their corresponding modules, and clinical features.

### 2.4 Identification of liver fibrosis–related mitochondrial genes and enrichment analysis

The intersection of three gene sets—differentially expressed genes (DEGs), genes from WGCNA modules significantly associated with liver fibrosis, and the mitochondrial gene set—was taken to obtain liver fibrosis–related mitochondrial genes (25). A Venn diagram was used for visualization. Subsequently, the selected genes were subjected to Gene Ontology (GO) and Kyoto Encyclopedia of Genes and Genomes (KEGG) pathway enrichment analyses using the clusterProfiler package to explore their potential biological processes and signaling pathways.

### 2.5 Transcription factor analysis

Transcription factor (TF) activity was inferred using the decoupleR package (26). The TF–target regulatory network was obtained from the CollecTRI database, which integrates 12 different resources, providing broad coverage and improved accuracy in identifying perturbed TFs compared to DoRothEA and other literature-based GRNs. In addition, CollecTRI assigns weights to TF–target interactions according to their regulatory mode (activation or repression). The Univariate Linear Model (ULM) method implemented in decoupleR was applied to fit a linear model for each sample based on TF–gene regulatory weights, with the *t*-value of the slope representing the TF activity score. A positive value indicates TF activation, whereas a negative value indicates repression. Finally, significantly dysregulated TFs were identified and visualized.

### 2.6 Machine learning model construction and validation

The intersection genes obtained from differential expression analysis, WGCNA modules and the mitochondrial gene set were used as model inputs. To ensure unbiased sample allocation and reproducibility, the primary dataset was partitioned into training (70%) and testing (30%) subsets using a stratified random split, thereby maintaining equal proportions of control and fibrosis samples in both subsets. Within the training set, 10-fold cross-validation was performed to optimize model performance, and a grid search was applied to tune key hyperparameters. Model performance was evaluated on the held-out test set using the area under the ROC curve (AUC) and the confusion matrix.

For external validation, the independent dataset GSE167216 was used, with model generalization ability assessed by calculating AUC and plotting ROC curves (21). In addition, the dataset GSE119953, which contains multiple etiologies of liver fibrosis including cholestatic, metabolic and alcoholic injury, was incorporated as an extended validation cohort to further examine cross-context robustness. AUC values and gene expression patterns were evaluated to assess model performance in this cohort.

To interpret the decision-making process of the model, the SHAP (SHapley Additive exPlanations) algorithm was applied to compute the contribution of each feature to the prediction outcome. This yielded a global ranking of feature importance, and the major predictive genes were visualized through SHAP summary plots.

### 2.7 Cell–cell interaction analysis

Cell–cell communication analysis was performed using the CellChat package, with the CellChatDB.mouse database serving as the reference (27).

### 2.8 Cell culture and siRNA-mediated knockdown of ACOT9

The human hepatic stellate cell line LX-2 was obtained from the Shanghai Institute of the Chinese Academy of Sciences. Cells were cultured in Dulbecco’s Modified Eagle Medium (DMEM; Gibco) supplemented with 10% fetal bovine serum (FBS; Gibco) and 1% penicillin–streptomycin, and maintained in a humidified incubator at 37 °C with 5% CO_2_. LX-2 cells at passages 4–6 were used for all experiments.

For ACOT9 knockdown, siRNA oligonucleotides targeting human ACOT9 (siACOT9) and a non-targeting control siRNA (siNC) were purchased from GenePharma (Shanghai, China). Three siRNA sequences targeting ACOT9 were designed and screened, and the most effective sequence (si-ACOT9-1) was selected for subsequent experiments. The siRNA sequences used in this study were as follows (5′ → 3′): siACOT9 sense, CCACCAUACAUGAGAUGUUTT; siACOT9 antisense, AACAUCUCAUGUAUGGUGGTT; siNC sense, UUC UCC GAA CGU GUC ACG UTT; siNC antisense, ACG UGA CAC GUU CGG AGA ATT.

LX-2 cells were seeded into 12-well plates at a density of 1.0 × 10^5^ cells per well and incubated overnight to reach approximately 50–70% confluence. siRNA–lipid complexes were prepared according to the manufacturer’s protocol and added to the cells for transfection.

Cells were collected 48 hours after transfection for qRT-PCR to assess ACOT9 mRNA knockdown, and for Western blot analysis to evaluate ACOT9 protein knockdown and downstream fibrosis-related markers including α-SMA, COL1A1, and TGF-β. Both siNC and mock-transfection controls were included. Knockdown efficiency was confirmed at both the mRNA and protein levels. All experiments were performed with at least three biological replicates.

### 2.9 Human clinical sample collection and animal experiments

Human liver tissue samples were obtained from patients with liver cancer undergoing surgical resection at Gongli Hospital of Shanghai Pudong New Area. The study protocol was approved by the Medical Ethics Committee of Gongli Hospital of Shanghai Pudong New Area (Approval No.: GLYY1s2025-070), and written informed consent was obtained from all participants prior to surgery. The collected samples comprised two groups: the normal control group, obtained from paracancerous normal liver tissues distant from the tumor focus; and the fibrosis group, acquired from non-tumorous paracancerous regions exhibiting evident fibrotic lesions, which were also located distal to the tumor.

A total of 50 male C57BL/6 mice (6–8 weeks old, weighing 20–24 g) were purchased from SPF (Suzhou) Biotechnology Co., Ltd. (Suzhou, China) and housed under specific pathogen-free (SPF) conditions. Prior to the experiments, the mice were acclimated for 3 days in a controlled environment with a temperature of 22 ± 1 °C, humidity of 55 ± 5%, and a 12-h light/dark cycle. Sterile food and water were provided ad libitum. All animal experiments were conducted in strict accordance with the Guide for the Care and Use of Laboratory Animals (NIH, USA) and were approved by the Institutional Animal Care and Use Committee of Shanghai Novopathway Biotechnology Co., Ltd. (Approval No.: P0820240921A).

The mice were randomly assigned into two groups (n = 10 per group): a CCl_4_-induced fibrosis group and a vehicle control group. Liver fibrosis was induced in the CCl_4_ group by intraperitoneal injection of carbon tetrachloride (CCl_4_) diluted 1:1 in olive oil at a dose of 2 mL/kg body weight, administered once every 3 days for 8 weeks. The control group received intraperitoneal injections of an equal volume of olive oil at the same time points.

To evaluate the translational potential, an independent experimental cohort was established. This cohort consisted of 30 mice in total, randomly assigned into three groups (n = 10 per group). The induction protocols for the Normal Control and CCl_4_-induced fibrosis groups were completely identical to those described above. For the Celastrol-treated fibrosis group, Celastrol (purity ≥ 98%; Sigma-Aldrich, Cat# C0869, USA) was initially dissolved in dimethyl sulfoxide (DMSO) and subsequently diluted with sterile phosphate-buffered saline (PBS) to achieve a final DMSO concentration of < 0.1% (v/v). Mice in this group received daily intraperitoneal injections of Celastrol at a dose of 0.5 mg/kg body weight, starting from the 5th week of CCl_4_ modeling until the end of the 8-week experimental period.

### 2.10 Histopathological examination (HE and Masson staining)

Liver tissues were fixed in 10% neutral buffered formalin for 24–48 h, paraffin-embedded, and sectioned at 4 μm thickness. For Hematoxylin and Eosin (HE) staining, nuclei were stained with hematoxylin, differentiated with acid alcohol, blued, counterstained with eosin, dehydrated through graded ethanol, cleared in xylene, and mounted with neutral resin.

For Masson’s trichrome staining, nuclei were stained with Weigert’s iron hematoxylin, cytoplasm with Ponceau fuchsin–acid fuchsin, collagen was differentiated with phosphomolybdic/phosphotungstic acid, stained with aniline blue (or light green), terminated with acetic acid, dehydrated, and mounted.

### 2.11 Immunofluorescence double staining (TSA method)

Paraffin-embedded liver sections were dewaxed three times with an environmentally friendly deparaffinization reagent (10 min each), followed by rehydration through 100%, 95%, and 75% ethanol (5 min each) to distilled water. Antigen retrieval was performed in EDTA buffer (pH 9.0) using high-pressure heating (timed 1.5 min after pressure release), cooled to room temperature, and washed with TBST. Endogenous peroxidase activity was blocked with 3% H_2_O_2_ for 10 min, and nonspecific binding was blocked with 10% goat serum at 37 °C for 30 min. Primary antibodies were incubated overnight at 4 °C, followed by HRP-conjugated secondary antibody incubation (1:4000, 37 °C for 45 min). Signal amplification was achieved using Cy5-tyramide (1:400, with 0.003% H_2_O_2_, for 10 min). The first signal was stripped by microwave treatment in citric acid buffer for 5 min, after which the sections were re-blocked, incubated with the second primary antibody (1:800, overnight at 4 °C), and subjected to the same HRP-conjugated secondary antibody treatment. A second TSA labeling was performed using iFluor 488 (1:600, 10 min). Nuclei were counterstained with DAPI (1:500) for 5 min. Finally, the sections were mounted with antifade reagent, stored at 4 °C in the dark, and imaged using a fluorescence microscope.

The following primary antibodies were used: PCK2 (Proteintech Group, Inc., Cat. No. 14892-1-AP, 1:100); ACOT9 (Proteintech Group, Inc., Cat. No. 15901-1-AP, 1:100); ALDH1B1 (Proteintech Group, Inc., Cat. No. 15560-1-AP, 1:100); TOM20 (Proteintech Group, Inc., Cat. No. 11802-1-AP, 1:400, 1:800); COL1A1 (E8F4L, CST, XP #72026, 1:4000); TGF-β (Immunoway, Cat. No. YS0004, 1:2000); and Goat Anti-Rabbit IgG H&L (HRP) (Abcam, ab205718, 1:4000).

### 2.12 Western blot analysis

LX-2 cells or liver tissue samples were lysed in RIPA buffer supplemented with protease and phosphatase inhibitor cocktails. The lysates were centrifuged at 12,000 × g for 15 min at 4 °C, and the supernatants were collected. Protein concentrations were determined using a BCA Protein Assay Kit.

Equal amounts of protein (20–40 µg per lane) were mixed with 5× SDS loading buffer and heated at 95 °C for 5 min. Proteins were separated by SDS–polyacrylamide gel electrophoresis (SDS-PAGE) and transferred onto PVDF membranes. Membranes were blocked with BSA blocking solution for 1 h at room temperature and then incubated overnight at 4 °C with primary antibodies, including ACOT9 (Proteintech, 15901-1-AP, 1:1000), ALDH1B1 (Proteintech, 15560-1-AP, 1:1000), PCK2 (Proteintech, 14892-1-AP, 1:1000), α-SMA (Abcam, ab124964, 1:1000), COL1A1 (Abcam, ab260043, 1:1000), TGF-β (Abcam, ab179695, 1:1000), and β-actin (Proteintech, 20536-1-AP, 1:5000).

After washing, membranes were incubated with HRP-conjugated secondary antibodies for 1 h at room temperature. Protein signals were visualized using an enhanced chemiluminescence (ECL) detection kit and imaged with an Amersham Imager 600 system. Band intensities were quantified using ImageJ software and normalized to β-actin.

### 2.13 Statistical analysis

All statistical analyses were conducted using R software (version 4.3.3) and GraphPad Prism 10. For comparisons between two groups, a two-tailed unpaired Student’s t-test was applied when data satisfied assumptions of normality and homogeneity of variance; otherwise, the Wilcoxon rank-sum test was used. For comparisons among multiple groups, one-way ANOVA followed by Tukey’s *post hoc* test was performed for normally distributed data, while the Kruskal–Wallis test was used for non-normally distributed data. A p-value < 0.05 was considered statistically significant. All quantitative experiments, including Western blot, qRT-PCR, and TSA-IF, were performed with three biological replicates.

## 3 Results

### 3.1 Identification of mitochondria-related differential genes associated with liver fibrosis

**Figure 1:**
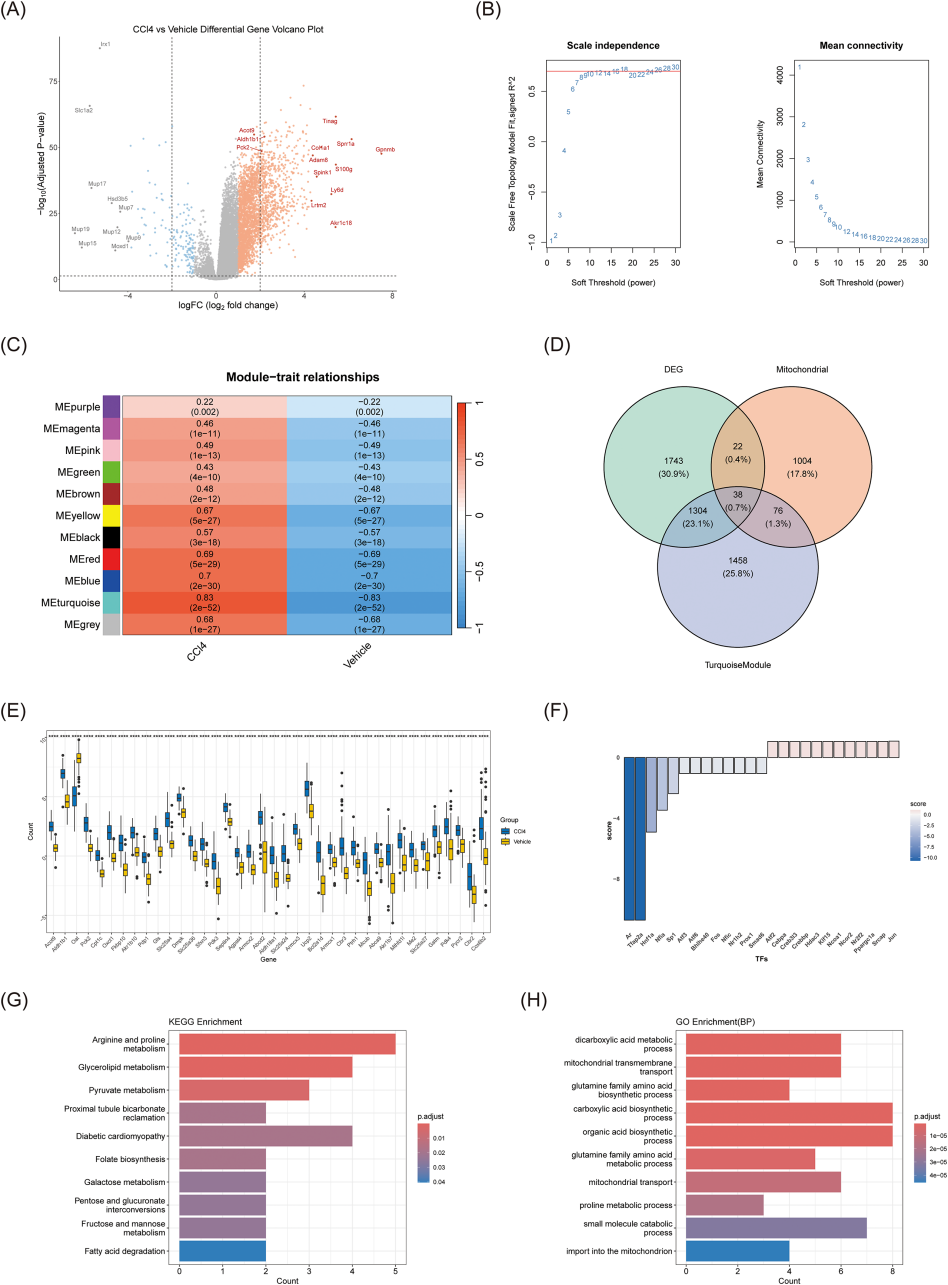
Identification of mitochondria-related candidate genes in liver fibrosis. **(A)** Volcano plot of differentially expressed genes (DEGs) between normal and fibrotic liver samples. **(B)** Soft-threshold power selection (power = 12) for weighted gene co-expression network analysis (WGCNA). **(C)** Module–trait heatmap showing the correlation between gene modules and liver fibrosis status. **(D)** Venn diagram of mitochondrial genes, DEGs, and fibrosis-associated WGCNA modules, yielding 38 intersecting genes. **(E)** Boxplots comparing the expression of the 38 intersecting mitochondrial genes between normal and fibrotic samples. **(F)** Transcription factors predicted to regulate the 38 intersecting genes. **(G)** KEGG pathway enrichment analysis of the 38 intersecting genes. **(H)** GO functional enrichment analysis of the 38 intersecting genes.

In the GSE152329 dataset, differential expression analysis using the limma package identified a total of 3,107 differentially expressed genes (DEGs), including 2,948 upregulated and 159 downregulated genes. Most of the DEGs were significantly upregulated in the CCl_4_-treated group compared to the baseline controls (**Figure 1A**). Subsequently, after detecting and removing outlier samples via hierarchical clustering (**Supplementary Figure 5A**), weighted gene co-expression network analysis (WGCNA) was performed using whole-transcriptome data to identify key gene modules highly correlated with liver fibrosis (**Figures 1B, C**). By intersecting the DEGs, the key module genes identified by WGCNA, and a curated mitochondrial gene set, a total of 38 mitochondria-associated genes relevant to liver fibrosis were obtained (**Figure 1D**). Except for Ocat, all of these genes were significantly upregulated in the CCl_4_ group (**Figure 1E**).

Upstream transcription factor analysis of these 38 genes revealed that androgen receptor (AR) and TFAP2A were significantly enriched regulators, both showing decreased transcription factor activity scores in the liver fibrosis group (**Figure 1F**). To further delineate the specific regulatory landscape of our target gene, we constructed a co-expression network. Our results demonstrated that specific regulators, notably *Atf3*, *Hdac3*, and *Smad6*, exhibited a strong co-expression correlation with *Acot9* (**Supplementary Figure 5B**).

Functional enrichment analysis indicated that the KEGG pathways were primarily involved in amino acid, lipid, and energy metabolism (including arginine and proline metabolism, glycerolipid metabolism, pyruvate metabolism, and fatty acid degradation), carbohydrate metabolism (such as galactose metabolism, fructose and mannose metabolism, and pentose and glucuronate interconversions), one-carbon metabolism (folate biosynthesis), as well as disease-related pathways such as proximal tubule bicarbonate reclamation and diabetic cardiomyopathy (**Figure 1G**).

Gene Ontology (GO) analysis of biological processes (BP) further revealed enrichment in dicarboxylic acid, organic acid, and carboxylic acid biosynthetic processes, mitochondrial transmembrane and overall transport, glutamine family amino acid metabolism and biosynthesis, proline metabolic processes, small molecule catabolic processes, and mitochondrial import (**Figure 1H**).

### 3.2 Key gene selection via machine learning and functional enrichment analysis

**Figure 2:**
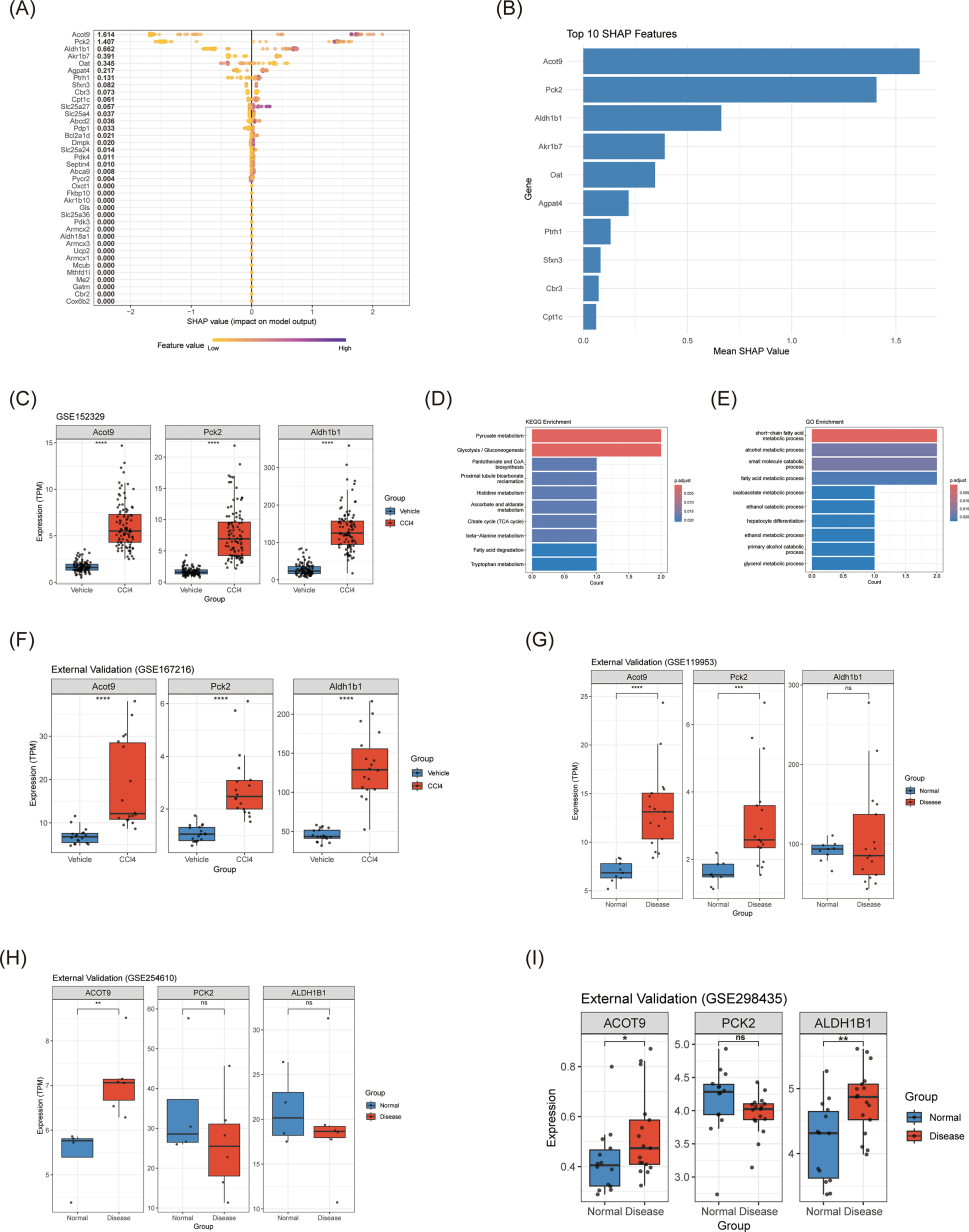
Machine learning-based identification and validation of key mitochondrial genes in liver fibrosis. **(A)** SHAP dot plot showing the ranking of 38 candidate genes based on their contribution in the XGBoost model. **(B)** Bar plot of feature importance, highlighting the top 10 ranked genes. **(C)** Boxplots comparing the expression of Acot9, Aldh1b1, and Pck2 between normal and fibrotic liver samples. **(D)** KEGG pathway enrichment analysis of Acot9, Aldh1b1, and Pck2. **(E)** GO functional enrichment analysis of Acot9, Aldh1b1, and Pck2. **(F)** Boxplots comparing the expression levels of Acot9, Aldh1b1, and Pck2 between normal and fibrotic liver samples (GSE167216). **(G)** Boxplots comparing the expression levels of Acot9, Aldh1b1, and Pck2 between normal and fibrotic liver samples (GSE119953). **(H)** Clinical validation in the human dataset GSE254610. Boxplots comparing the expression of ACOT9, ALDH1B1, and PCK2 between healthy donors and patients with liver cirrhosis. **(I)** Clinical validation in an independent human dataset GSE298435. Boxplots comparing the expression of ACOT9, ALDH1B1, and PCK2 between healthy donors and patients with liver cirrhosis. ***P < 0.001, **P < 0.01, *P < 0.05, ns, not significant.

Among the 38 mitochondria-related differential genes, we employed the XGBoost algorithm to prioritize critical features associated with the fibrotic phenotype, and applied SHAP (SHapley Additive exPlanations) for interpretability analysis (**Figures 2A, B**). According to global importance ranking based on the mean absolute SHAP values, the top three features—Acot9, Aldh1b1, and Pck2—were identified as key candidate targets. Boxplots demonstrated that all three genes were significantly upregulated in the CCl_4_-treated group compared to baseline controls (**Figure 2C**). SHAP value distributions further confirmed that high expression levels of these genes were strongly associated with the “CCl_4_-treated” status, consistent with their upregulation in the fibrotic group.

KEGG enrichment analysis revealed that these genes were primarily involved in pyruvate metabolism and glycolysis/gluconeogenesis pathways, while GO enrichment analysis showed significant association with biological processes such as short-chain fatty acid metabolism, alcohol metabolic process, small molecule catabolic process, and fatty acid metabolism (**Figures 2D, E**).

### 3.3 Validation of expression patterns across independent datasets and species

To verify the reliability of the identified targets, external validation was first performed using the independent mouse dataset GSE167216. After data preprocessing consistent with the discovery cohort, all three genes—Acot9, Aldh1b1, and Pck2—exhibited a consistent upregulated trend in the CCl_4_-treated group compared to baseline controls (**Figure 2F**). This confirms that the aberrant expression of these mitochondrial genes is a stable feature of CCl_4_-induced liver fibrosis (**Supplementary Figure 4**).

To further assess the robustness of these targets across different etiologies, an additional validation was conducted using the dataset GSE119953, which comprises multiple fibrosis models including cholestatic, metabolic, and alcoholic injury. Consistent with our primary findings, Acot9 and Pck2 were markedly upregulated in fibrotic samples compared with controls. However, Aldh1b1 showed no significant difference between groups in this multi-etiology cohort (**Figure 2G**). These results suggest that Acot9 and Pck2 may represent more conserved targets across diverse fibrotic triggers.

Crucially, to evaluate clinical relevance, we analyzed two independent human transcriptomic datasets (GSE254610 and GSE298435). Notably, ACOT9 was found to be consistently and significantly upregulated in liver tissues from patients with cirrhosis compared to healthy controls across both cohorts (**Figures 2H, I**). This cross-species validation highlights ACOT9 as the most robust and clinically relevant mitochondrial target among the candidates, warranting further functional investigation.

### 3.4 Alterations in cellular composition and cell-type-specific expression of the three genes in a liver fibrosis model

**Figure 3:**
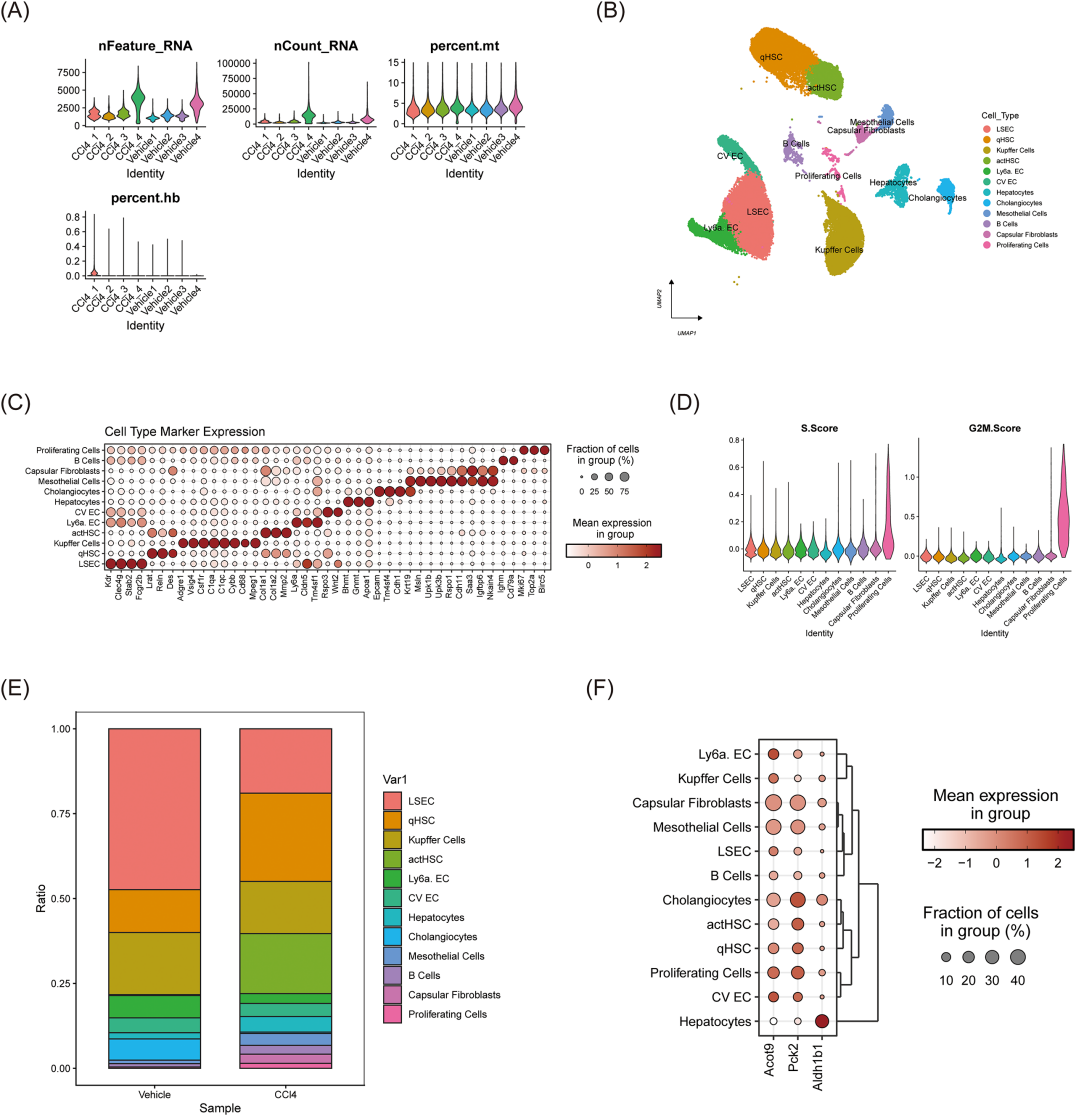
Single-cell transcriptomic analysis of liver fibrosis. **(A)** Quality control metrics before cell filtering, including the distribution of gene counts (nFeature_RNA), UMI counts (nCount_RNA), and the percentages of mitochondrial and hemoglobin genes across samples. **(B)** Cell clustering of liver fibrosis samples. **(C)** Cell-type annotation of single-cell RNA-seq data. **(D)** Cell cycle analysis of single-cell transcriptomic data. **(E)** Proportional changes of different cell types between normal and fibrotic groups. **(F)** Expression distribution of Acot9, Aldh1b1, and Pck2 across different cell types.

Following quality control, UMAP clustering and annotation of the integrated single-cell dataset identified a total of 12 distinct cell types (**Supplementary Figure 3**), including liver sinusoidal endothelial cells (LSEC), quiescent hepatic stellate cells (qHSC), Kupffer cells, activated hepatic stellate cells (actHSC), Ly6a-positive endothelial cells (Ly6a.EC), central vein endothelial cells (CV EC), hepatocytes, cholangiocytes, mesothelial cells, B cells, capsular fibroblasts, and proliferating cells (**Figures 3A–D**).

Compared to the baseline control, the CCl_4_-treated group exhibited marked alterations in cellular composition (**Figure 3E**), characterized by a reduced proportion of endothelial cell-related subsets (LSEC, Ly6a.EC, CV EC) and an increased proportion of both actHSC and qHSC. Additionally, an elevated proportion of hepatocytes and a decreased proportion of cholangiocytes were observed.

Spatial mapping based on feature gene expression (Feature, Dot, and Violin plots) revealed clear cell-type specificity of the three target mitochondrial genes (**Figure 3F**). Acot9 was predominantly enriched in Ly6a.EC and CV EC. Pck2 was mainly expressed in cholangiocytes, actHSC, and qHSC. Aldh1b1 was primarily distributed in hepatocytes.

### 3.5 Alterations in cell–cell communication in the CCl_4_-induced fibrosis model

**Figure 4:**
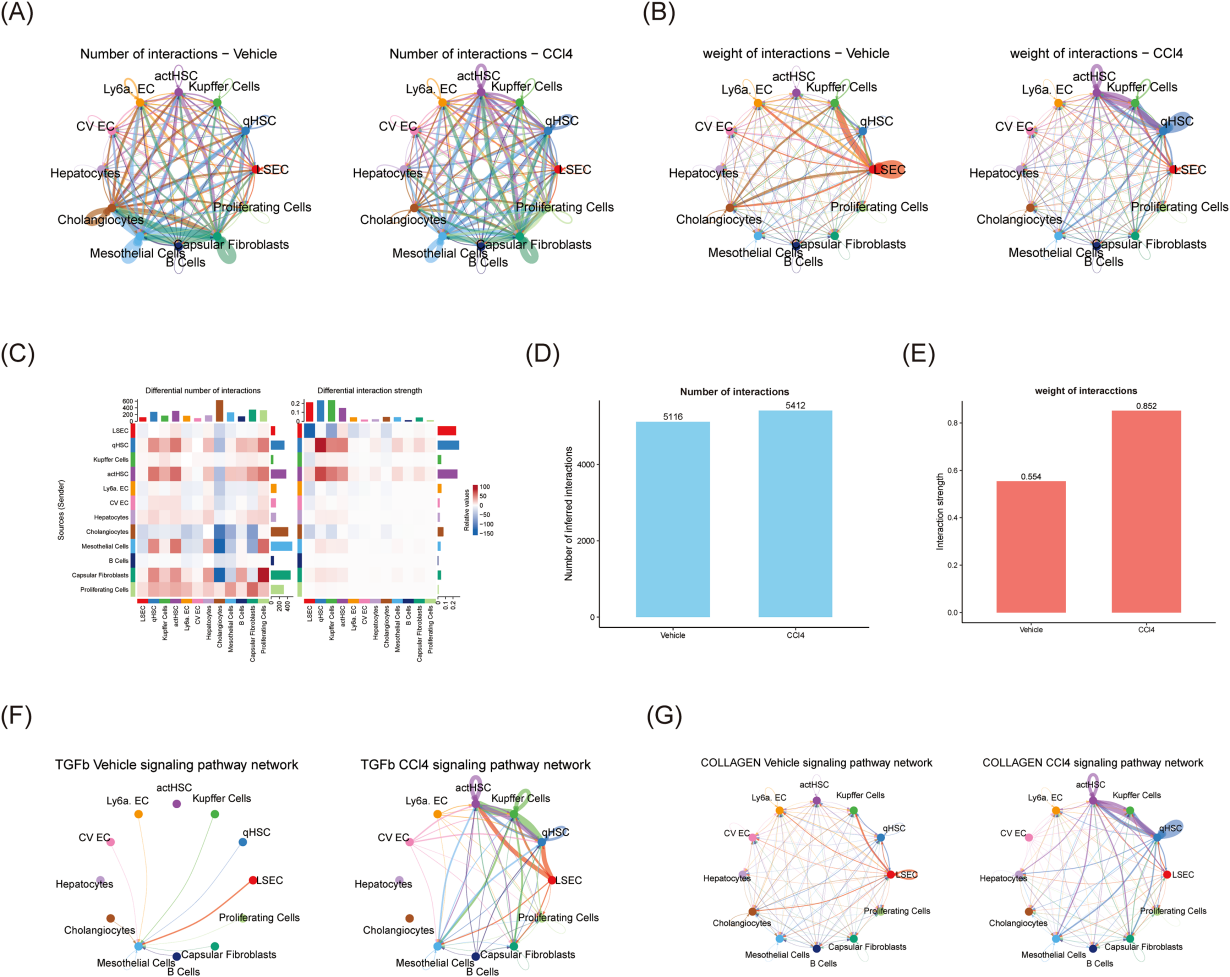
Cell–cell interaction analysis between normal and fibrotic groups. **(A)** Circos plot showing changes in the number of cell–cell interactions between normal and fibrotic groups. **(B)** Circos plot showing changes in the strength of cell–cell interactions between normal and fibrotic groups. **(C)** Differential analysis of the number and strength of cell–cell interactions, with red indicating enhancement in the fibrotic group compared with the normal group, and blue indicating reduction. **(D)** Bar plot quantifying the differences in the number of cell–cell interactions between normal and fibrotic groups. **(E)** Bar plot quantifying the differences in the strength of cell–cell interactions between normal and fibrotic groups. **(F)** Circos plot showing changes in interaction strength within the TGF-β signaling pathway between normal and fibrotic groups. **(G)** Circos plot showing changes in interaction strength within the COLLAGEN signaling pathway between normal and fibrotic groups.

Comparative analysis between the Vehicle and CCl_4_ groups using CellChat suggested notable differences in both the number of interactions and the distribution of interaction strengths. In terms of network topology, the communication “hubs” in the CCl_4_ group appeared to be predominantly concentrated among stromal and immune cells, with predicted increases in incoming and outgoing interaction degrees in actHSCs, qHSCs, and Kupffer cells. In contrast, endothelial-related subsets such as LSECs, Ly6a.ECs, and CV ECs showed reduced predicted contributions to the network, implying a potential shift in the central axis of intercellular signaling from endothelial dominance toward a stromal–immune axis during fibrosis progression (**Figures 4A–E**).

Differential interaction analysis further indicated that HSC-related outgoing and incoming signaling edges were predicted to be enhanced in the CCl_4_ group, involving a broad spectrum of target cells including Kupffer cells, hepatocytes, and proliferating cells. Conversely, interactions originating from endothelial subsets were predicted to decline (**Figure 4C**). This emerging pattern of “stromal/immune enhancement and endothelial attenuation” is consistent with known structural and functional remodeling of the fibrotic microenvironment.

At the signaling pathway level, both groups exhibited multiple canonical ligand–receptor pathways mediating cell–cell communication, such as COLLAGEN and TGFβ. For example, network analysis of the TGFβ pathway revealed that HSCs displayed relatively limited interactions in the Vehicle group, whereas in the CCl_4_ group, interactions among actHSCs, qHSCs, Kupffer cells, and LSECs were predicted to be strengthened. Similarly, the COLLAGEN signaling network showed substantial predicted remodeling in the CCl_4_ group, with greater interaction strength among actHSCs, qHSCs, and Kupffer cells compared to the Vehicle group (**Figures 4F, G**). Collectively, these analyses suggest that TGFβ and COLLAGEN signaling may play a key role in coordinating communication between stromal and immune cell compartments during fibrosis progression.

### 3.6 Experimental validation of mitochondrial gene expression and functional relevance

**Figure 5:**
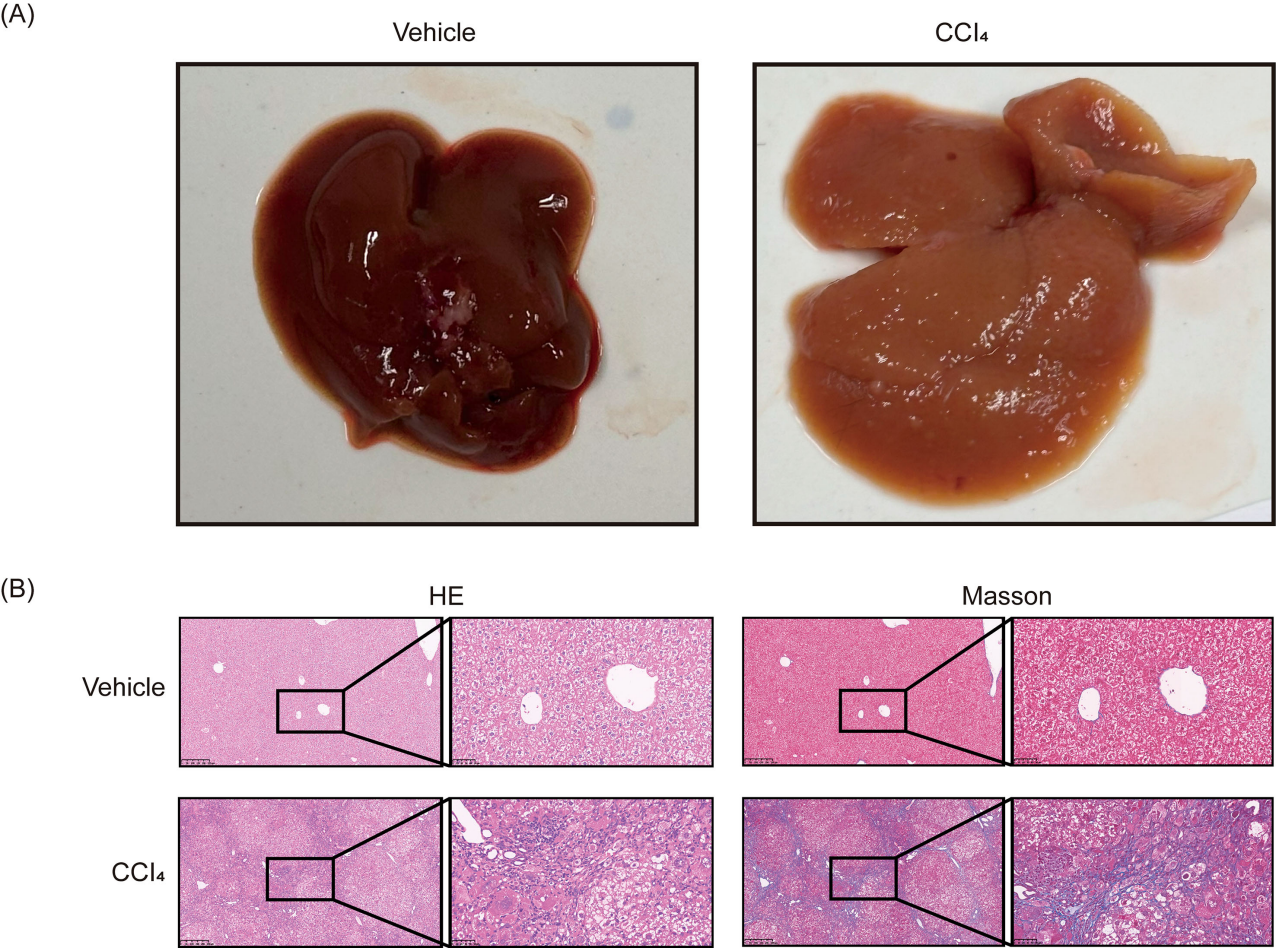
Histological and immunofluorescence validation in the liver fibrosis mouse model. **(A)** Representative gross images of livers from normal and CCl_4_-induced fibrotic mice. **(B)** Hematoxylin–eosin (HE) and Masson’s trichrome staining of liver tissues in normal and fibrotic groups.

**Figure 6:**
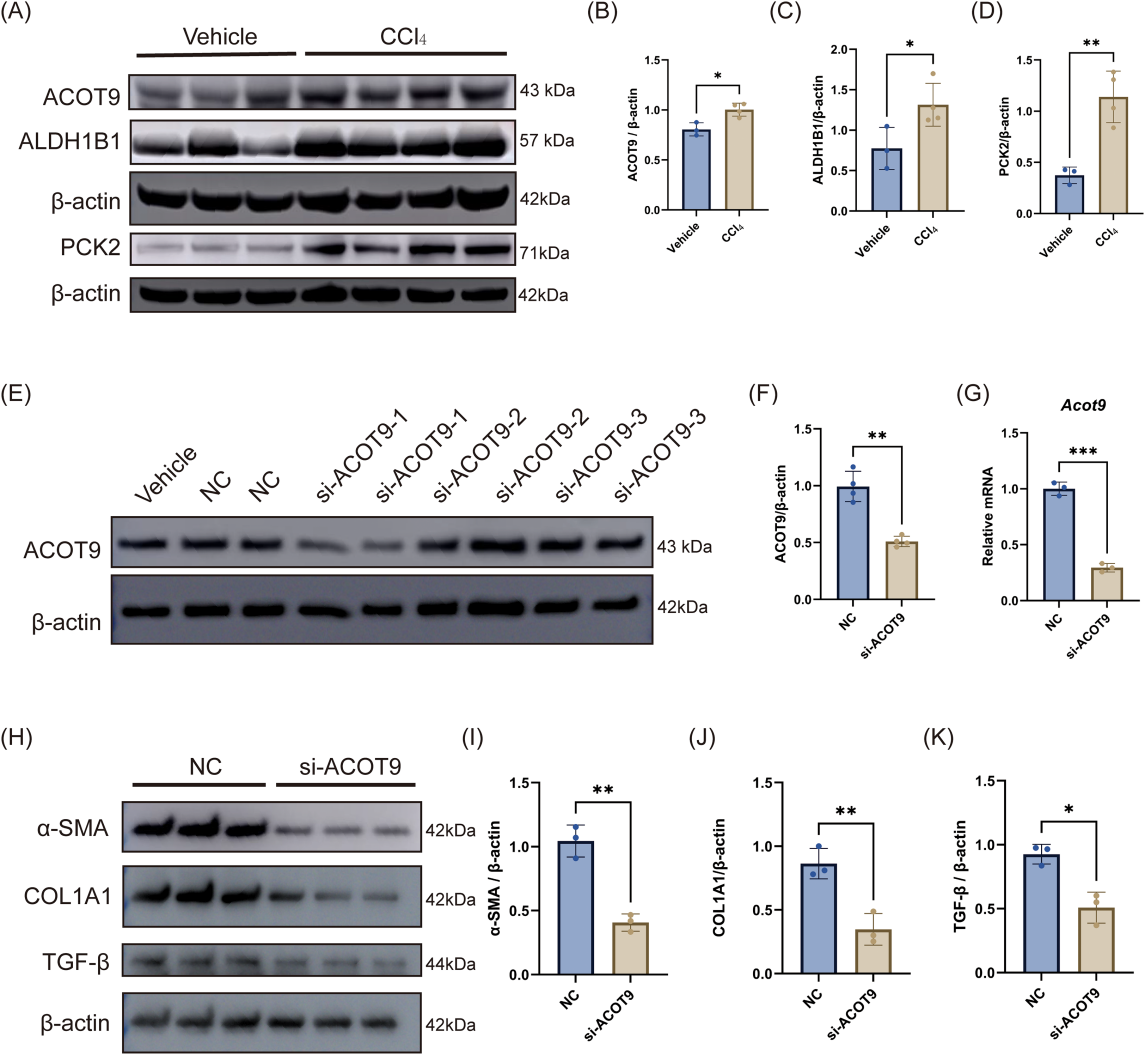
*In vivo* validation of mitochondrial candidate genes and *in vitro* functional assessment of ACOT9. **(A–D)** Western blot analysis showing increased protein expression of ACOT9, ALDH1B1, and PCK2 in CCl_4_-induced fibrotic liver tissues compared with control groups. Representative blot images and corresponding quantification are shown. **(E, F)** Western blot analysis confirming ACOT9 knockdown efficiency in LX-2 hepatic stellate cells, including representative blot images and quantitative analysis. **(G)** qRT-PCR analysis verifying the efficiency of ACOT9 mRNA knockdown in LX-2 cells. **(H–K)** Western blot analysis and quantification of α-SMA, COL1A1, and TGF-β in LX-2 cells following ACOT9 knockdown.

To further validate the findings from the above bioinformatic analyses, we established a mouse model of liver fibrosis and conducted *in vivo* experiments (**Figures 5A, B**). The results showed that the protein expression levels of all three mitochondrial-related genes—Acot9, Aldh1b1, and Pck2—were significantly increased in fibrotic liver tissues, fully consistent with their transcriptional expression patterns (**Figures 6A–D**). Immunofluorescence staining further confirmed the elevated expression of these genes within fibrotic regions, revealing their spatial distribution and cellular activation characteristics under pathological conditions (**Supplementary Figures 1A–D**). In addition, the expression levels of TGFβ and COL1A1, two key mediators of intercellular communication, were also markedly elevated in fibrotic tissues, providing additional *in vivo* support for the reliability of the bioinformatic predictions (**Supplementary Figure 2A**). Given that our single-cell analysis revealed ACOT9 is predominantly localized within hepatic stellate cells (HSCs) (**Supplementary Figure 5C**), we strategically selected the human HSC line LX-2 for subsequent *in vitro* functional assays. we next knocked down ACOT9 in LX-2 hepatic stellate cells and examined the protein expression of α-SMA, COL1A1, and TGFβ (**Figures 6E–G**). The results showed that ACOT9 knockdown significantly reduced the expression of these fibrosis-related proteins, indicating that changes in ACOT9 expression are associated with the fibrotic phenotype of hepatic stellate cells (**Figures 6H–K**).

### 3.7 ACOT9 expression is significantly upregulated in human liver fibrosis

**Figure 7:**
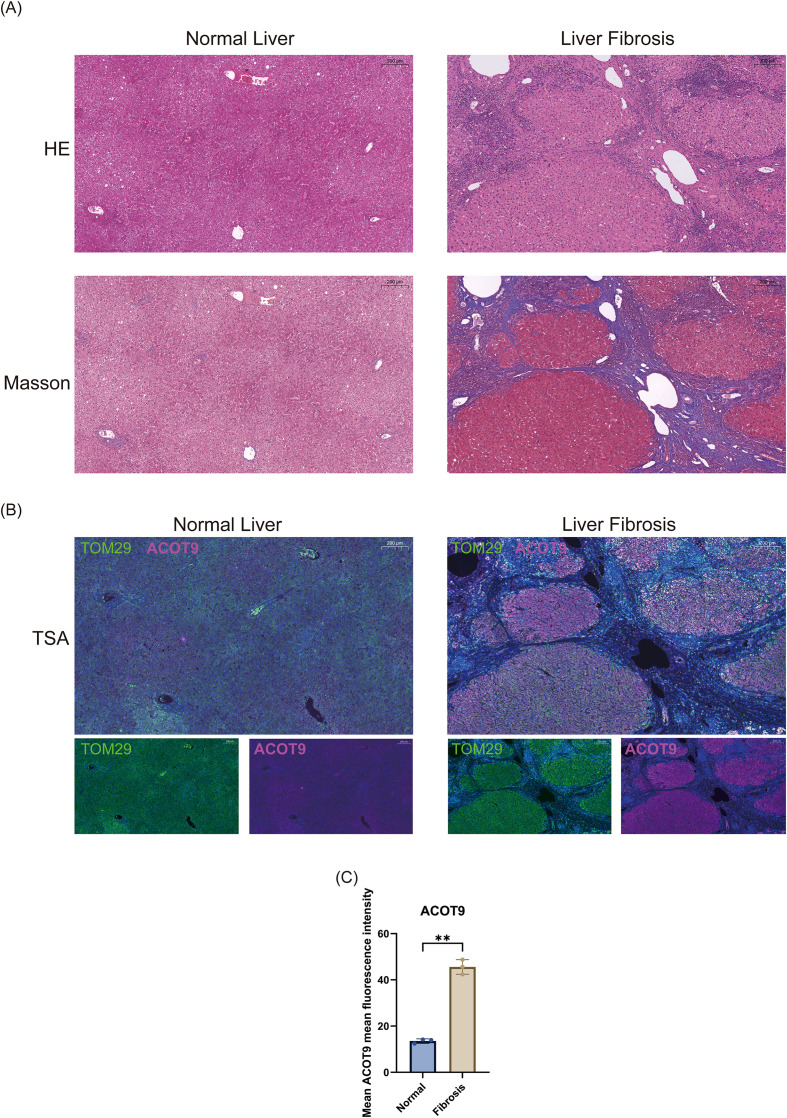
ACOT9 expression in human fibrotic liver tissues. **(A)** Representative images of H&E and Masson’s trichrome staining in human paracancerous normal liver tissues and non-tumorous paracancerous liver tissues with evident fibrosis. Scale bars, 200 μm. **(B)** Representative TSA-based immunofluorescence images showing the *in situ* expression of ACOT9 and TOM20 in human liver samples. Nuclei were counterstained with DAPI. Scale bars, 200 μm. **(C)** Quantitative analysis of the relative protein expression of ACOT9 based on fluorescence intensity. Data are presented as mean ± SD (n = 3 per group). Student’s t-test, *P < 0.05, **P < 0.01, ***P < 0.001.

To further corroborate our findings in a clinical context, we collected human liver clinical samples, including normal paracancerous tissues distant from the tumor and non-tumorous tissues with evident fibrotic lesions. H&E and Masson’s trichrome staining confirmed the structural disruption and collagen deposition in the fibrotic group (**Figure 7A**). Subsequently, we performed TSA-based immunofluorescence staining on these clinical samples. Consistent with our bioinformatic predictions, the protein expression of ACOT9 was significantly elevated in human fibrotic liver tissues compared to normal controls (**Figures 7B, C**).

### 3.8 Pharmacological intervention with celastrol suppresses ACOT9 expression *in vivo*

Given the critical role of ACOT9 in liver fibrosis, we further explored its translational potential as a therapeutic target. We established an independent *in vivo* pharmacological validation cohort comprising a Normal Control group, a CCl_4_-induced fibrosis group, and a Celastrol-treated group.

**Figure 8:**
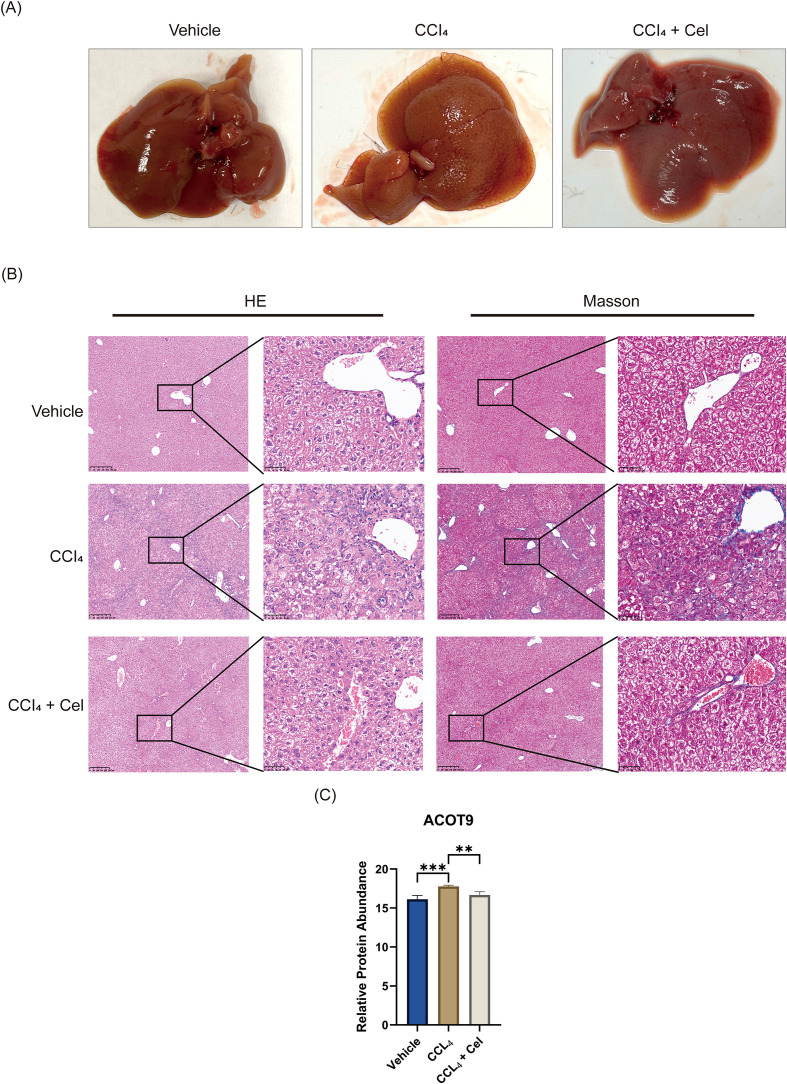
Pharmacological intervention with Celastrol alleviates liver fibrosis and suppresses ACOT9 expression *in vivo*. **(A)** Representative macroscopic morphological photographs of mouse liver tissues from the Normal Control, CCl_4_-induced fibrosis, and Celastrol-treated groups. **(B)** Representative images of H&E and Masson’s trichrome staining of mouse liver sections from the indicated groups, showing the extent of hepatic damage and collagen deposition. Scale bars, 250 μm. **(C)** Quantitative results of ACOT9 protein expression levels among the three experimental groups. Data are presented as mean ± SD (n = 10 per group). One-way ANOVA followed by Tukey’s *post hoc* test, *P < 0.05, **P < 0.01, ***P < 0.001.

Our morphological and histological evaluations revealed that Celastrol treatment effectively alleviated CCl_4_-induced liver damage and extracellular matrix deposition (**Figures 8A, B**). Importantly, to determine whether the anti-fibrotic effect of Celastrol was accompanied by alterations in ACOT9, we analyzed the protein expression profiles in the liver tissues of this parallel cohort. Proteomic analysis demonstrated that the protein level of ACOT9, which was markedly overexpressed in the CCl_4_-induced model group, was significantly downregulated following Celastrol intervention (*P* < 0.05) (**Figure 8C**).

## 4 Discussion

In this study, by integrating WGCNA, machine learning, and single-cell RNA sequencing, we identified three mitochondria-associated genes—ACOT9, ALDH1B1, and PCK2—that show strong associations with liver fibrosis. Instead of merely serving as statistical biomarkers, these genes appear to reflect broader metabolic disturbances characteristic of fibrotic progression, including alterations in mitochondrial lipid metabolism, oxidative stress responses, and cellular energy balance. Crucially, our cross-species validation highlighted ACOT9 as a conserved target upregulated in both murine models and human cirrhosis (exhibiting an AUC of 1.00 in the validation cohort), and its functional knockdown in hepatic stellate cells (HSCs) provided direct evidence of its regulatory role in fibrogenesis. Collectively, our findings provide hypothesis-generating insights into the metabolic landscape of liver fibrosis and highlight potential therapeutic targets for metabolic intervention.

The specific upregulation of ACOT9 indicates enhanced mitochondrial fatty acid turnover, a metabolic shift that has been reported to promote lipid biosynthesis and acetyl-CoA accumulation in liver disease models (28). Consistent with this, we observed that silencing ACOT9 in LX-2 cells significantly attenuated the expression of α-SMA, COL1A1, and TGF-β. This suggests that ACOT9 is not just a passive marker but an active driver of HSC activation, aligning with previous findings in NAFLD and HCC (28–31). Meanwhile, the increased expression of ALDH1B1 may represent a compensatory protective response aimed at detoxifying excessive aldehydes generated during fibrogenesis, thereby counteracting oxidative damage (32–34).

Although these three genes perform distinct biological functions, together they may outline a vicious cycle of “metabolic reprogramming and oxidative stress” within the fibrotic liver. Based on our findings and existing literature, we propose a hypothetical mechanistic model: initial hepatic injury leads to the upregulation of ACOT9, potentially driving abnormal lipid metabolism and excessive acetyl-CoA accumulation—a process known to generate high levels of reactive oxygen species (ROS). In this context, the upregulation of ALDH1B1 might represent a compensatory response aimed at detoxifying ROS and lipid peroxidation byproducts. Concurrently, the observed upregulation of PCK2 suggests an adaptive metabolic rewiring. Under conditions of mitochondrial stress and lipid dysregulation, elevated PCK2 may serve to sustain gluconeogenesis or cataplerosis, thereby fulfilling the high demand for biosynthetic precursors (such as amino acids for collagen production) required during fibrogenesis. However, this sustained state of high metabolic flux likely exacerbates mitochondrial load, ultimately leading to cellular homeostasis failure and the release of DAMPs, which sustain the activation of HSCs. Therefore, ACOT9 may function as a pivotal node deeply embedded in this pathological network (35).

Our single-cell RNA sequencing data, together with CellChat analysis, offer preliminary clues regarding how these mitochondrial genes may be involved in fibrotic microenvironment changes. The cell-type–specific expression patterns imply that metabolic alterations may occur in different cell populations (e.g., ACOT9 in endothelial subsets and HSCs) yet ultimately influence fibrosis through intercellular interactions. In particular, the strengthened TGF-β and COLLAGEN signaling networks observed in our study may serve as critical downstream effectors of the metabolic dysregulation described above.

Given the regulatory role of ACOT9 in HSC activation identified in our study, targeting mitochondrial metabolic reprogramming represents a promising therapeutic strategy. Current anti-fibrotic therapies largely focus on blocking downstream TGF-β signaling or collagen cross-linking. However, targeting upstream mitochondrial drivers like ACOT9 could potentially cut off the energy supply and metabolic substrates required for myofibroblast transdifferentiation. Future drug discovery efforts could focus on developing specific small-molecule inhibitors of ACOT9 to evaluate their efficacy in reversing established fibrosis.

This study also has several limitations. First, utilizing a predefined list of mitochondria-related genes introduces selection bias, potentially omitting novel unannotated differentially expressed genes (DEGs). Second, although we validated ACOT9 expression in a human cirrhosis dataset, further validation in large-scale, multicenter cohorts is still required. Furthermore, the upstream regulatory network of ACOT9 awaits further experimental verification.

To address these limitations, future studies will dissect the precise mechanisms linking ACOT9-mediated lipid metabolism to TGF-β signaling using multi-omics approaches. We plan to conduct *in vivo* validation using *Acot9* knockout mice and further investigate the upstream transcription factors of ACOT9. Additionally, we will evaluate the clinical diagnostic value of ACOT9 in patient serum or extracellular vesicles. Ultimately, these efforts aim to advance the clinical translation of ACOT9 as a novel therapeutic target and diagnostic tool for liver fibrosis.

## 5 Conclusion

In conclusion, this study integrates transcriptomic analysis and experimental validation to characterize the dysregulation of mitochondrial genes, specifically Acot9, Aldh1b1, and Pck2, in liver fibrosis. Our findings demonstrate that ACOT9 is consistently upregulated across species and contributes to hepatic stellate cell activation. These results suggest that ACOT9, along with the identified metabolic signature, may represent a potential target for therapeutic intervention, warranting further mechanistic investigation.

## Data Availability

The original contributions presented in the study are included in the article/**Supplementary Material**. Further inquiries can be directed to the corresponding author/s.
