# Supplementary material for: Identification of mitochondria-related biomarkers in liver fibrosis via interpretable machine learning and WGCNA: transcriptomic analysis and In Vivo validation
Source: Front Immunol. 2026 May 28;17:1705706. doi: 10.3389/fimmu.2026.1705706 (PMC13253275; doi:10.3389/fimmu.2026.1705706)
Supplement: Supplementary file 1 [file DataSheet1.pdf]

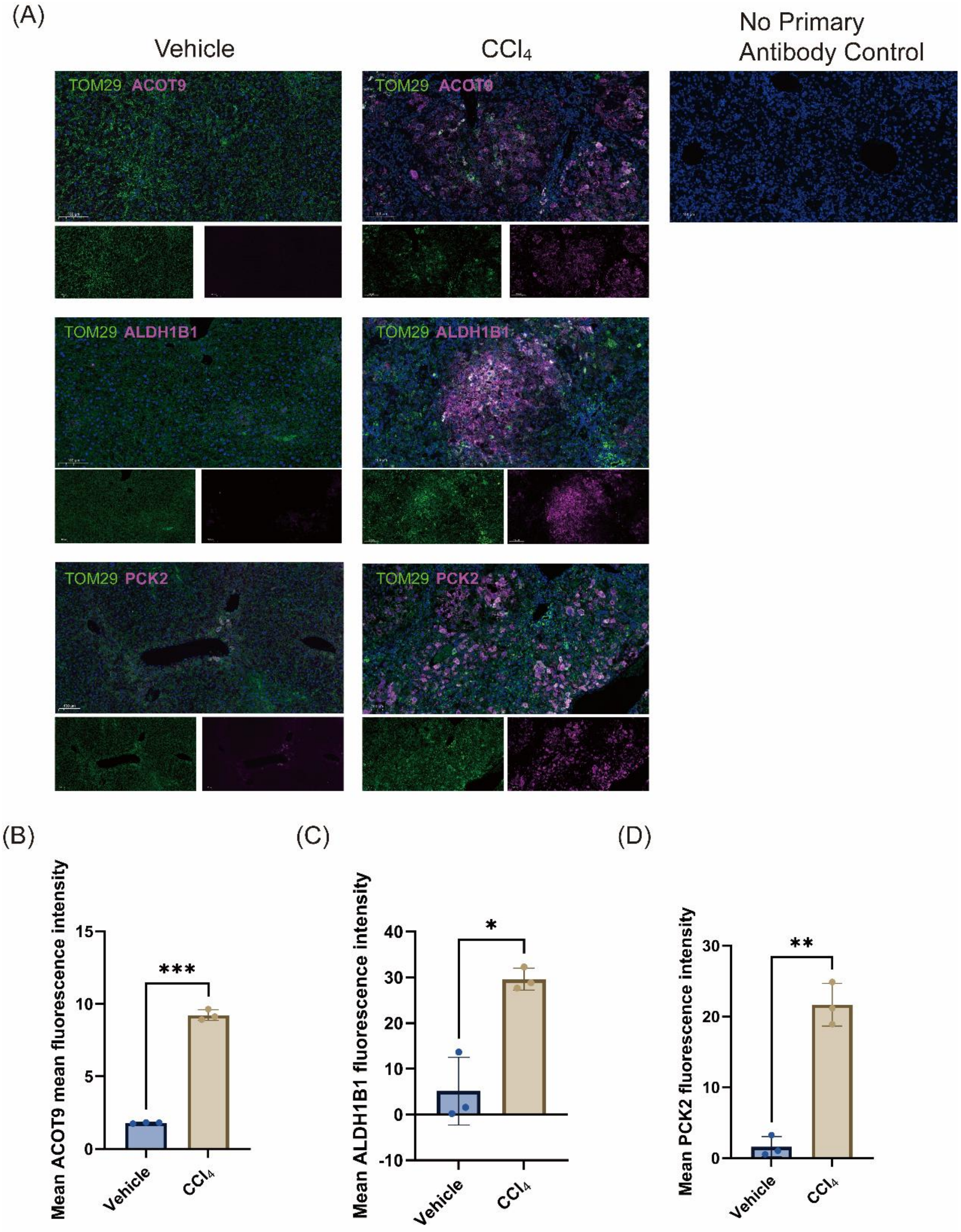

Fig. S1. Histological and immunofluorescence validation in the liver fibrosis mouse model.

(A) Immunofluorescence staining images showing the co-localization of TOM20 with ACOT9, ALDH1B1, and PCK2 in liver sections from normal and CCl_4-induced fibrotic mice.

(B) Quantification of fluorescence intensity for ACOT9.

(C) Quantification of fluorescence intensity for ALDH1B1.

(D) Quantification of fluorescence intensity for PCK2.

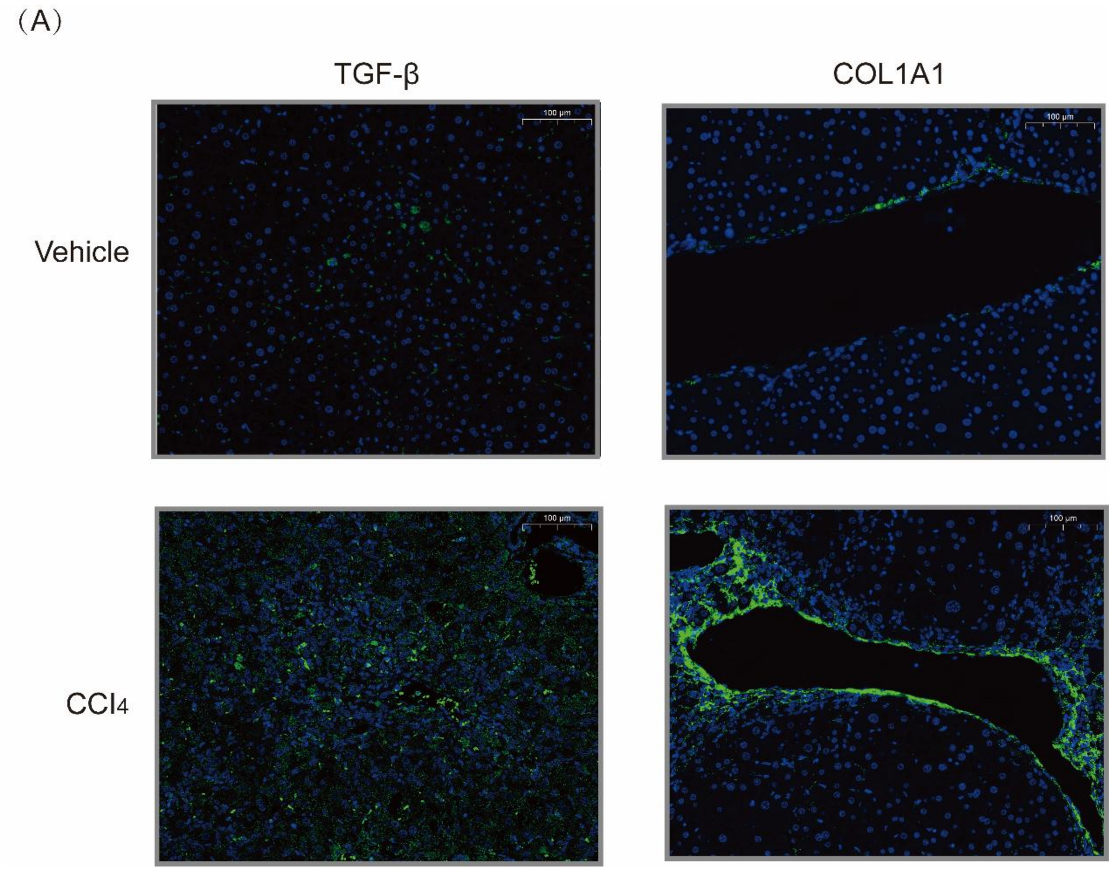

Fig. S2. Immunofluorescence validation of key fibrotic signaling molecules.

(A) Representative immunofluorescence staining of TGF-β and Col1a1 in liver tissues from normal and fibrotic mice.

(A)

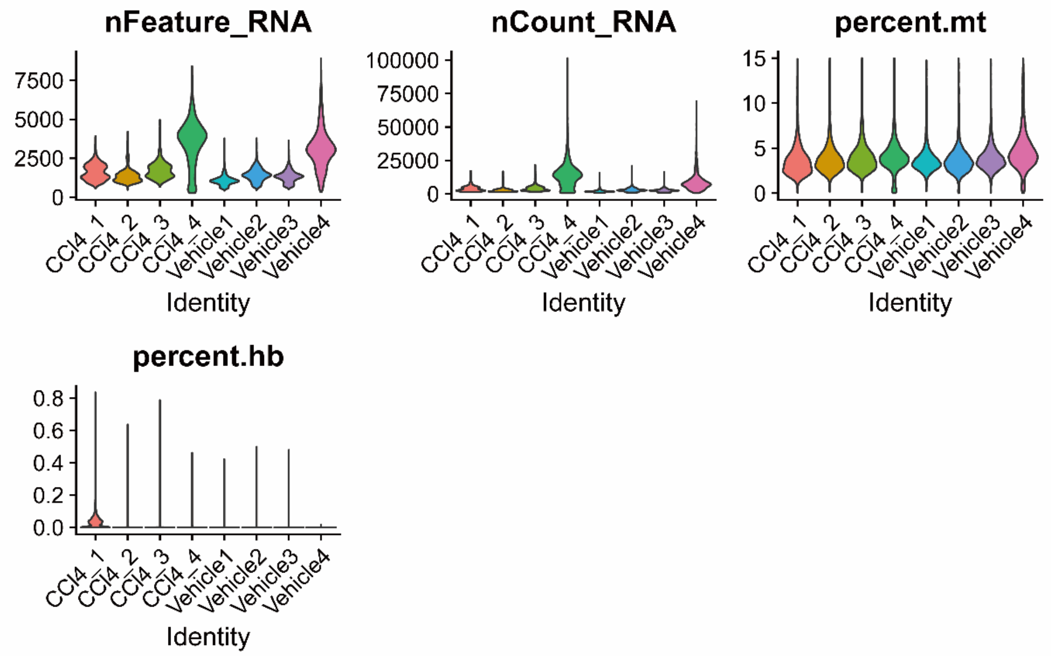

(B)

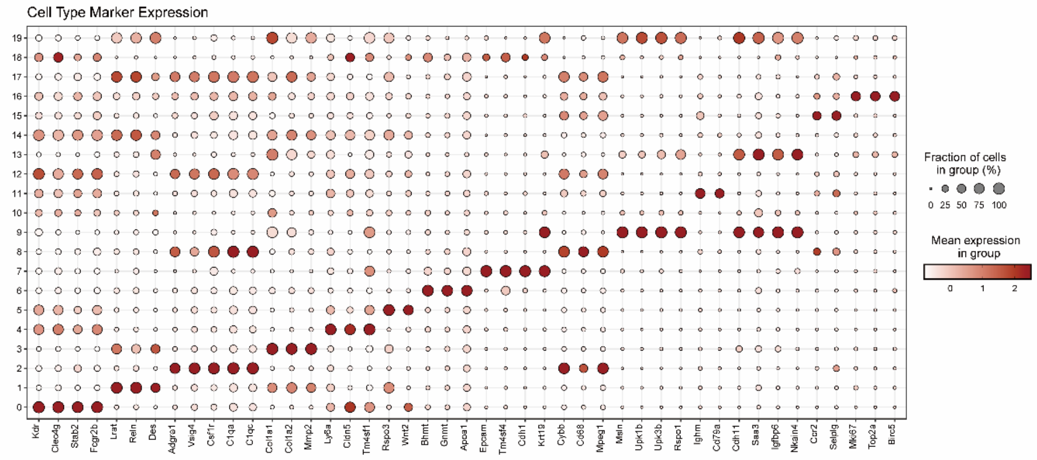

Fig. S3. Quality control assessment and pre-filtering clustering of single-cell transcriptomic data.

(A) Quality control metrics before cell filtering, including the distribution of gene counts (nFeature_RNA), UMI counts (nCount_RNA), and the percentages of mitochondrial and hemoglobin genes across samples.

(B) Original clustering plot prior to excluding the cell subpopulation that simultaneously expressed marker genes from two distinct cell types, which was used to identify and label doublet cell clusters for removal.

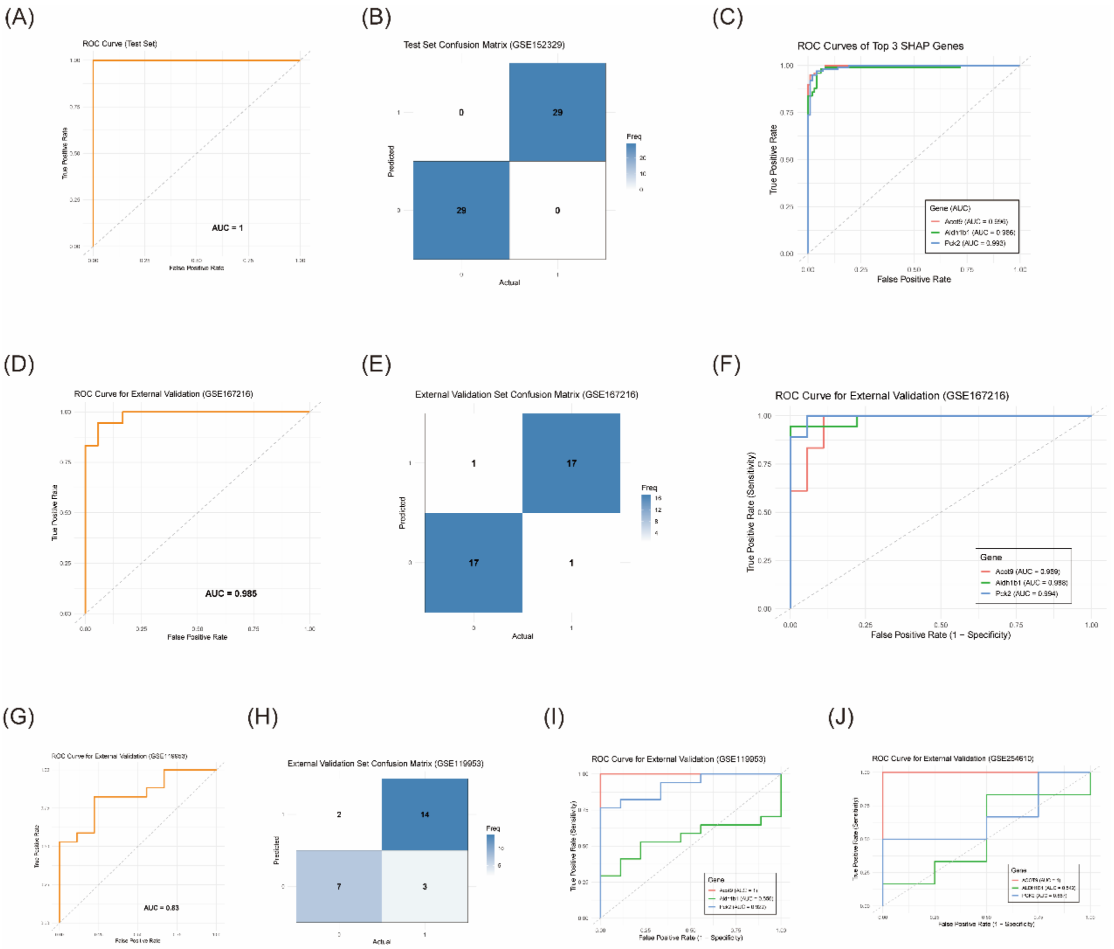

Fig. S4. Evaluation of the discrimination ability of the identified mitochondrial targets across multiple independent datasets.

(A–C) Performance evaluation in the testing dataset (GSE152329). (A) ROC curve of the three-gene combination in the testing dataset. (B) Confusion matrix of the three-gene combination in the testing dataset. (C) ROC curves of individual genes (Acot9, Aldh1b1, and Pck2) in the testing dataset.

(D–F) Validation in the external CCl_4-induced fibrosis dataset (GSE167216). (D) ROC curve of the three-gene combination in the external dataset (GSE167216). (E) Confusion matrix of the three-gene combination in the external dataset (GSE167216). (F) ROC curves of individual genes (Acot9, Aldh1b1, and Pck2) in the external dataset (GSE167216).

(G–I) Validation in the multi-etiology external dataset (GSE119953). (G) ROC curve of the three-gene combination in the external dataset (GSE119953). (H) Confusion matrix of the three-gene combination in the external dataset (GSE119953). (I) ROC curves of individual genes (Acot9, Aldh1b1, and Pck2) in the external dataset (GSE119953).

(J) Validation in the human cirrhosis dataset (GSE254610). (J) ROC curves evaluating the discrimination ability of individual genes (ACOT9, ALDH1B1, and PCK2) in the human dataset.

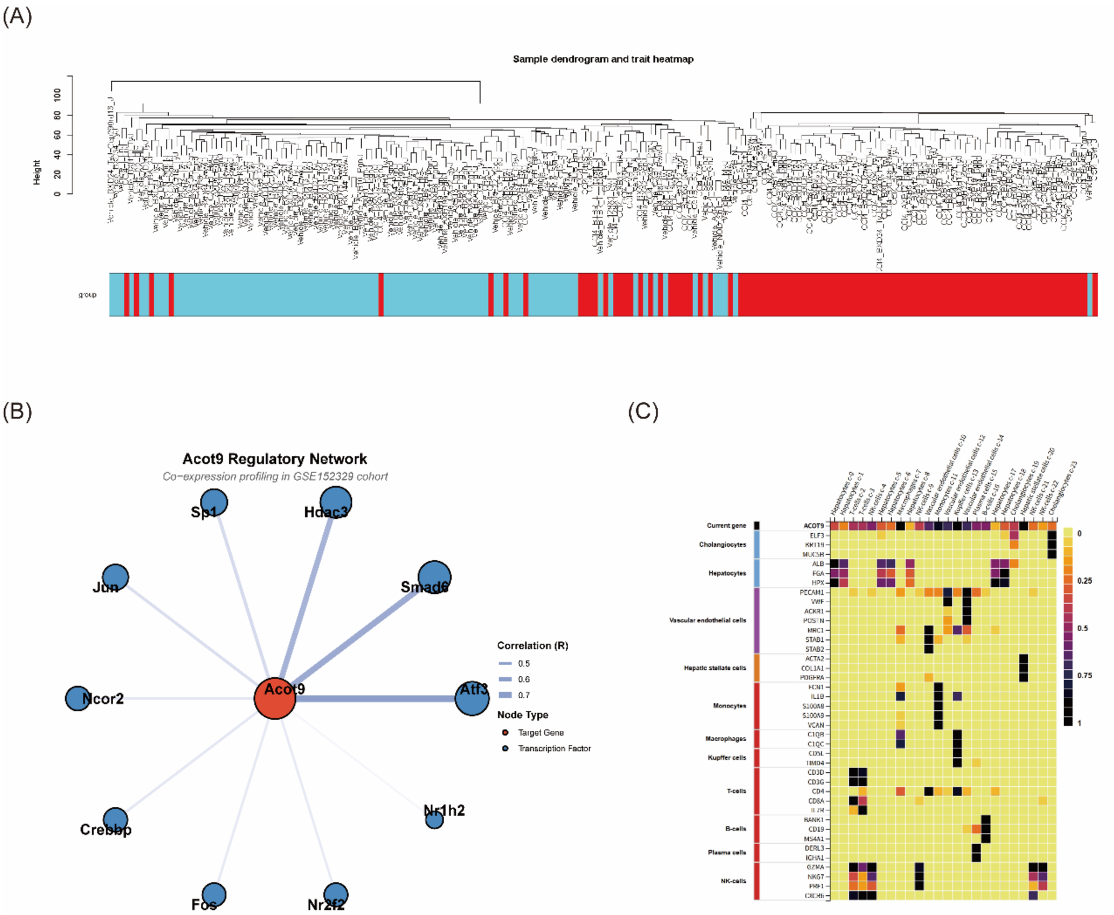

Fig. S5. Supplementary bioinformatic analyses of ACOT9.

(A) Sample clustering dendrogram constructed for the detection and removal of outlier samples prior to Weighted Gene Co-expression Network Analysis (WGCNA).

(B) Single-cell RNA-sequencing (scRNA-seq) analysis illustrating the expression distribution of ACOT9 across distinct cell populations in the human liver.

(C) Co-expression network analysis showing the potential regulatory relationships between *Acot9* and its associated transcription factors (TFs).
